# Use of Different O/W or W/O Emulsions as Functional Ingredients to Reduce Fat Content and Improve Lipid Profile in Spanish Cured Processed Meat Product (‘Chorizo’)

**DOI:** 10.3390/foods13142262

**Published:** 2024-07-18

**Authors:** Elena Martínez, Wagner Goncalves Vieira Júnior, Manuel Álvarez-Ortí, Adrián Rabadán, José Emilio Pardo

**Affiliations:** 1 Escuela Técnica Superior de Ingeniería Agronómica y de Montes y Biotecnología, Campus Universitario s/n, 02071 Albacete, Spain; elena.martinez@uclm.es (E.M.); manuel.alvarez@uclm.es (M.Á.-O.); jose.pgonzalez@uclm.es (J.E.P.); 2 Graduate Program in Agricultural and Livestock Microbiology, School of Agricultural and Veterinarian Sciences, São Paulo State University (UNESP), São Paulo 14884-900, Brazil; vieira.jr@unesp.br

**Keywords:** recovery waste, reformulated meat products, consumer preference, functional food, oil emulsion, dry cured sausages

## Abstract

In this work, three out of five types of oil-in-water and water-in-oil emulsions were selected to replace pork backfat to reduce the fat content and the improve monounsaturated and polyunsaturated fatty acid content in dry cured sausage (‘chorizo’). Different characteristics of the new product were studied: the texture profile, color, nutritional value, lipid profile, vitamin E and thiobarbituric acid (TBA) and sensory qualities. The use of emulsions to replace the animal fat affected all color parameters, obtaining darker, less red and yellow products, which could impact the consumer’s purchase intention. This replacement also altered the texture parameters, increasing or decreasing the hardness in comparison with the control sample. The cohesiveness, however, decreased in all cases, which meant that when the samples are cut for consumption, they disintegrated more than the traditional ones. The most relevant aspect for nutritional value is that the use of the new emulsions helped to reduce the total fat and energy value of the reformulated samples. The most significant aspect is that this reformulation helped to reduce the fat content—specifically, saturated fat—while increasing the content of Omega 3/6. The new formulas contained significant quantities of TBA and vitamin E when comparing them with the traditional product.

## 1. Introduction

Chorizo is a traditional Spanish processed raw meat product that is, however, available and consumed throughout the world [1]. In Spanish legislation, chorizo is defined as “sausages made from meat and fat, generally pork, although they may also be produced from meat and fat from other animals, coarsely or finely minced, and subjected to a curing process. Paprika is added as a characterizing ingredient, although other spices, seasonings, ingredients, and additives may be added. The mixture is kneaded and stuffed into natural or artificial casings, then undergoing a curing-maturing process, with or without fermentation. Optionally, they may be smoked, which gives them a typical aroma and flavor” [2]. 

The high quality of the protein, minerals and vitamins found in meat means that it is considered an important food for humans, but the excessive consumption of processed meat products is not recommended due to their high levels of sodium and fat [3]. The food industry faces the great challenge of seeking new formulations to replace saturated fatty acids and to do so without changing consumer habits. Vegetable oils have thus gained importance in recent years due to the benefits that they confer on the final products, as they have a positive impact on the nutritional composition by lowering the cholesterol content and enhancing the fatty acid profiles [4]. Their inclusion, however, is not always technologically feasible due to their taste, color and free fatty acid composition [5]. 

To substitute animal fat, this study used oils obtained from chia and pumpkin seeds. Pumpkin seeds were used not only for their benefits but also because they are considered agri-food waste. Chia seeds are high in fat (30–35%), carbohydrates (25–41%), primarily dietary fiber (non-digestible cellulose, pentosans and lignin) (18–30%), protein (20–22%), vitamins (A, B, K, E, D), minerals and antioxidants. The main fatty acids are linolenic acid (55–65%), linoleic acid (15–25%), oleic acid (6–8%) and stearic and palmitic acids (5–10%). Due to its high percentage of these fatty acids, which are beneficial for health, chia oil has become one of the most valuable in the world market. Many authors have suggested that, given its high percentage of α-linolenic acid, it could be used as an alternative source of ω-3 fatty acids for vegetarians and individuals allergic to fish [6]. 

Meanwhile, pumpkin seeds are known to have many health benefits. They are a natural source of phytosterols, antioxidants and vitamins such as tocopherols and carotenoids and an excellent source of unsaturated fatty acids, which constitute 98% of the seeds’ total fatty acids: palmitic acid (9.5–14.5%), steric acid (3.1–7.4%), oleic acid (21–46.9%) and linoleic acid (35.6–60.8%) [7]. These compounds are considered to be physiologically beneficial for the prostate gland, as well as presenting other benefits, such as being antiparasitic in the intestine [8].

When reformulating meat products, it should be borne in mind that animal fat has a unique texture, juiciness and flavor, contributing to their sensory acceptability. Previous studies have shown that directly adding vegetable and/or marine oils in liquid form negatively impacts the texture and sensory properties. However, the incorporation of oil-in-water emulsions can significantly enhance the textural properties of sausages, making them more tender and juicier [9]. This is primarily due to the ability of emulsions to retain water and fat, which contributes to a better mouthfeel and reduced cooking loss [10]. However, the use of emulsions must overcome challenges such as physical instability and the susceptibility to oxidation [11,12]. With the aim of solving these problems, scientists have investigated how to introduce oleogels or emulsions to reformulate meat products. These emulsions can be formulated by incorporating gelling agents from proteins, polysaccharides or a mixture of both, so that the polymers can interact, creating three-dimensional networks, increasing the water and oil retention capacity of meat products and reducing the possibility of rapid oxidation. 

Previous studies conducted by the scientific community focus on how to resolve or minimize the effect of replacing animal fat with vegetable fat on the texture, concluding that emulsified oils with structuring agents are those that confer similar characteristics to solid fats. The aim of the present work is thus to study the behavior of emulsions before their incorporation into the product and how they behave once the final product is obtained, focusing not only on physical analyses but also on nutritional, lipid and sensory analyses.

## 2. Materials and Methods

### 2.1. Raw Materials

The lean meat was acquired already minced, while we minced the pork backfat using a knife mill (Verder Scientific GmbH & Co. KG, Haan, Germany). Both ingredients were provided by “El Conchel”, a meat company (El Ballestero, Albacete, Spain). The chia seeds were purchased at a local store and the pumpkins seeds were provided by a fresh fruit and vegetable company, Vicente Peris (Albuixet, Valencia, Spain).

Once received, the pumpkin seeds were manually cleaned and dried at a temperature of 50 °C, using a standard oven (Heraeus UT6, Hanau, Germany), to reduce their moisture content to a maximum of 7%.

To facilitate oil extraction, we milled the raw material. For this purpose, a knife mill was used (Verder Scientific GmbH & Co. KG, Haan, Germany), in which we placed 250 g of seeds for grinding at 10,000 rpm for 10 s. Subsequently, the ground seeds were placed on cloths that served to retain the oil cake.

A hydraulic press (MECAMAQ Model DEVF 80, Vila-Sana, Lleida, Spain) was used to extract the oil from the chia and pumpkin seeds. The extraction conditions were 200 bar pressure and 10 min of extraction. After the oil was extracted, it was centrifuged using a CENTRONIC-BL (Croydon, UK) at a speed of 12,000 rpm for 5 min, to remove any impurities that might interfere with the subsequent analytical measurement. The oils were refrigerated at 4 °C in opaque glass bottles to avoid their exposure to light.

### 2.2. Oil Emulsion Formulation

With the aim of achieving a texture as similar as possible to that of pork backfat, using the chia and pumpkin oils, 5 types of emulsions were formulated, which, as well as providing stability, were also economical and simple to use. Table 1 presents the different formulations of the emulsions.

To produce the first emulsion (E1), the oil was heated to 60 °C, to dissolve the beeswax, following the manufacturer’s instructions. Beeswax has a melting point ranging between 62 and 64 °C and has a cream color. When the wax was completely dissolved in the oil, the mixture was poured over water and xanthan gum was added. To obtain the emulsion, it was stirred with the help of an electric blender for 5 min.

To produce the second emulsion (E2), we mixed all of the ingredients at the same time. An electric blender was used to texturize the mixture, mixing it for 3 min, until the consistency and color of the mixture changed.

For the third emulsion (E3), to obtain a homogenous mixture, the ingredients were mixed together for 4 min, using an electric blender. Then, the oil was poured into water.

For the fourth emulsion (E4), as in the previous cases, all ingredients were placed together and blended to a homogeneous mixture with the aid of a blender.

To produce the fifth emulsion (E5), the water and emulsifiers were mixed with an electric blender and the mixture was allowed to stand for 24 h. After the resting time, the previous mix was poured into oil, mixing with an electric blender for at least 7 min.

All emulsions were stored and refrigerated for 24 h at 4 ± 2 °C in translucent polypropylene glasses, covered with a piece of plastic wrap between the sample and the plastic cover, to avoid possible surface drying. After this time, they were used immediately.

In order to determine the emulsions that were most similar to animal fat, their texture was measured using a back extrusion test, carried out in a TA-TX Plus (Stable Micro Systems, Godalming, UK), measuring the cohesiveness, consistency, firmness and index of viscosity. Before performing the test, the samples were tempered in a stove for 1 h at 30 °C, and four measurements of each emulsion were taken. The best results were obtained with E2, E3 and E5, which, henceforth and depending on the type of vegetable oil used, will be referred to as described in Table 2.

### 2.3. Meat Product Reformulation

**Figure 1:**
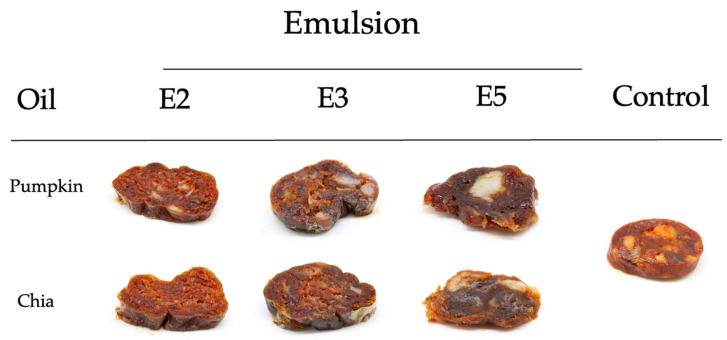
‘Chorizo’ formulas used in the present work.

To produce the chorizo (Figure 1), we used lean meat (66.3%); spices, namely garlic powder, paprika, salt and niter salt (5.3%); and fat (28.4%). We used pork backfat in the control, and, for the reformulated samples, the animal fat was fully replaced with the selected experimental emulsions, formulated with chia and pumpkin oils.

All formulations were produced at the same time and left to stand for 2 h in the fridge at 4 °C to homogenize the mixtures. To obtain the mixtures, all ingredients were mixed at the same time in a mixer. The mixture of all of the ingredients was stuffed into the casings with the help of a manual sausage stuffer. Each sausage was separated by tying it with a string at each end, with the weight being approximately 200 g per sausage. The formulations selected were the result of previous trials and preliminary tests carried out with a small number of consumers.

All chorizo samples produced were stored at 16 ± 2 °C and humidity of around 70%, to then be cured for 20 days. They were subsequently cut into 1.5 mm slices using a meat slicer, which were then vacuum-packed for their optimal conservation until the analyses were conducted.

### 2.4. Physical Analysis

#### Color

The color of the chorizo samples was determined using a Minolta CR-300 colorimeter (Minolta Camera Co., Ltd., Osaka, Japan). The color parameters were measured by reflection in five random areas of the chorizo in each batch. The illuminant used was D_65_. The tristimulus values obtained were used to calculate the CIELAB chromatic coordinates L* (relative lightness), a* (red–green component) and b* (yellow–blue component), following the recommendations of the International Commission on Illumination [13]. 

#### Texture

To determine the texture of the emulsions, we used a TA-XT Plus texture analyzer (Stable Micro Systems, Godalming, UK). We selected the re-extrusion test to obtain values for firmness, consistency, cohesiveness and the viscosity index. The conditions used were an assay speed of 1 mm/s and a distance of 50 mm. The accessory consisted of a 35 mm disc and a 50 kg load cell.

To measure the texture of the cured sausages, a texture profile analysis (TPA) was performed and the same texturometer was used as for the emulsions, albeit this time equipped with a 50-mm-diameter probe at 3.3 mm/s^−1^. The hardness, cohesiveness, elasticity and masticability were determined.

### 2.5. Proximate Analysis

The proximal composition analysis was conducted at the Service of Analysis and Innovation in Products of Animal Origin, located on the campus of the University of Extremadura in Cáceres (Spain). All samples were shipped fresh and vacuum-packed.

In the nutritional analysis, we determined the moisture content, protein, total fat, total carbohydrates, crude fiber and energy value. The moisture content was measured in an oven, placing the sample at 100 °C for 24 h until a constant weight was obtained. Meanwhile, the protein content was determined using the Kjeldahl method, multiplying the nitrogen content by a conversion factor of 6.25. Fat was estimated gravimetrically using the filter bag technique, after extracting the sample with petroleum ether in an Ankom XT10 extraction system. To determine the fiber content, we applied the Weende technique, adapted to the filter bag, determining the resulting organic residue after digestion with sulfuric acid and sodium hydroxide solutions using an Ankom 220 fiber analyzer (Ankom, Macedon, NY, USA). The carbohydrates were obtained by subtracting the sum of the content of protein, fat, water and ash from the total weight of the sample. Finally, the energy values were calculated using the relative content of protein, fat and carbohydrates, applying the Atwater factors of 4 kcal/g for protein, 9 kcal/g for fat and 4 kcal/g for carbohydrates.

### 2.6. Fatty Acid Profile, Vitamin E Determination and Thiobarbituric Acid

The fatty acid and vitamin E analyses were conducted at the Service of Analysis and Innovation in Products of Animal Origin, located on the campus of the University of Extremadura in Cáceres (Spain). All samples were shipped fresh and vacuum-packed.

The lipid profile, vitamin E content and thiobarbituric acid (TBA) index were determined following the methodology described in Martínez et al. (2023) [5]. These were obtained by gas chromatography, using a Shimadzu GC-2010 Plus chromatograph (Shimadzu, Tokyo, Japan). We also used a fused-silica capillary column CPSil 88 (50 m × 0.25 mm) with a film thickness of 0.20 m (Varian, Middelburg, The Netherlands) and helium as a carrier gas (120 kPa). Each fatty acid methyl ester (FAME) was identified by direct comparison with a standard mixture (FAME 37, Supelco, Bellefonte, PA, USA). Two samples from each batch were analyzed after fat extraction, with the results being expressed as a percentage of each FAME.

### 2.7. Sensory Evaluation

To evaluate the consumer acceptance of the samples, an affective test was chosen. Such tests measure the subjective reaction to the product. Specifically, we implemented the “Test for Measuring the Degree of Satisfaction”, which uses verbal hedonic scales. The test was carried out in the sensory analysis laboratory at the Higher Technical School of Agricultural and Forestry Engineering and Biotechnology, on the Albacete campus of the University of Castilla–La Mancha (UCLM).

The samples were marked with randomly chosen keys, being evaluated by 101 consumers, who assessed their external appearance, texture, smell and taste. Each hedonic description was assigned a 9-point scale (−4: I dislike it very much, 0: I neither like it nor dislike it, +4: I like it very much) [14]. The samples were served in slices and the vacuum bags were opened 3 h beforehand to aerate and temper the samples. To evaluate the external appearance, the samples were placed on a separate tray, where the consumers evaluated the individual samples as they would be presented on the market. The odor, flavor and texture were assessed on the tasting table.

### 2.8. Statistical Analysis

All experiments were performed at least twice, expressing the results as means and standard deviations. Significant differences were determined by analysis of variance (ANOVA), with a 5% level of significance, and by Tukey’s test (*p* < 0.05). All statistical analyses were performed using the SPSS program, version 23.0, for Windows.

## 3. Results and Discussion

### 3.1. Texture of Oil Emulsions

Table 3 shows the results obtained for the texture parameters (firmness, consistency, cohesiveness and viscosity index) of the five emulsions produced, with the aim being to select the three most similar to that of the pork backfat used as the control.

Emulsions differ depending on the formulation, since the interaction between the emulsifying agent and the emulsion may affect the overall perception of the food [15], as well as the sources of texturization, which, according to their concentrations, can vary the physical factors [15,16]. In the reformulation of a product, the least possible variation in the characteristics of the standard product is always sought, to avoid disapproval or reclassification as a new product [17,18]. In this sense, we selected the emulsions by prioritizing their proximity to the control treatment.

The emulsion based on HPMC and xanthan gum (E5) obtained the values closest to those of the pork backfat (control), being statistically the same with the exception of the consistency. Emulsions E2 (based on guar gum and inulin) and E3 (based on defatted chia flour and inulin) were those that obtained the values closest to pork backfat with respect to the consistency parameter. The firmness and consistency parameters indicated the solid properties of the emulsion, while cohesiveness and viscosity indicate its liquid properties [19]. In conclusion, emulsions E2, E3 and E5 were those selected for the reformulation of chorizo.

### 3.2. Physical Characteristics of Different ‘Chorizo’ Formulas

By using the oil emulsions selected in the study of the texture of the proposed oil emulsion, six chorizo samples were formulated. The physical parameters assessed in these chorizo samples were the color and texture. Table 4 shows the results for the color parameters (L*: relative lightness; a*: red–green component; b*: yellow–blue component; C*: chroma or purity).

The color of food plays a key role in choice and has a significant impact on the perception of taste and the final attractiveness of the product. It is thus an element that directly affects consumer preferences [20]. 

The traditionally produced cured chorizo was found to present higher values for all color parameters considered. High relative lightness (L*), typical of white colors, was clearly shown in the control cured chorizo, prepared with pork backfat. When this animal fat was replaced with vegetable oils, the values for this parameter decreased, resulting in darker cured chorizo. This observation is supported by other articles reporting the loss of lightness when animal fat is replaced with vegetable oils, also highlighting the correlation between the ingredient preparation process and the colorimetric parameters [21,22]. The decrease in lightness relative to the control sample may also be related to the pigments that replace the animal fat, known to provide lighter tones [23].

The red–green component (a*) was the parameter of maximum interest in colorimetric terms, since cured chorizo belongs to the red line of meat products, characterized by the addition of paprika (red in color) in its preparation. The control sample was that with the reddest color, differing significantly from the rest of the reformulations. This is consistent with a previous study, which reported that, in a meat emulsion with added vegetable oil, the red–green component decreased in comparison to the control sample [21].

Our control sample also presented higher values for parameter b* (yellow–blue component), indicating a stronger yellow tone. The yellowing decreased due to the presence of certain vegetable pigments found in chia seed and pumpkin seed oils [5,24]. 

The lowest a* and b* values were found in the emulsion using HPMC and xanthan gum, which might have been due the transparency of HPMC, which did not directly affect the emulsion [25]. 

Texture is also a key parameter in determining food quality. Table 5 shows the texture parameters of the different chorizo formulations produced.

The use of the emulsifier based on defatted chia flour and inulin produced cured chorizo of a harder, more elastic and chewy texture. According to [26], the addition of vegetable oils may increase the hardness of foods, as the vegetable oil fat globule is smaller than that of animal fat, resulting in stronger protein–protein and protein–lipid interactions.

The emulsion using HPMC and xanthan gum yielded the lowest values for all texture parameters evaluated, despite using the same vegetable oils as the other emulsions. Adding HPMC to foods is correlated with a decrease in hardness, causing a softening effect [27,28,29]. In contrast to other materials, such as inulin, which can increase the hardness [30], as also found in our study. The emulsion with HPMC and xanthan gum also presented lower elasticity, statistically at the same level as the sample, and masticability.

Cohesiveness was the most negative texture parameter for the new reformulations, being much lower than that found for the control sample, which might lead to the greater disintegration of the ingredients at the time of consumption.

### 3.3. Nutritional Composition

Nutritional analysis is key in understanding the chemical composition and nutritional quality of foods, yielding important data. The results of this analysis for the cured chorizo are presented in Table 6.

When analyzing the nutritional composition of foods, a significant reduction in total fat content, in general, and saturated fats, in particular, is positively valued [31,32]. This can be achieved by substituting pork backfat with vegetable oil emulsions, in which water is added, leading to lower fat content [23]. It is worth noting that saturated fats are associated with several health risks [33] and reducing them thus helps to produce healthier foods.

An excess of saturated fatty acids, common in animal fats, can cause inflammatory processes and muscle degradation, as well as being linked to a higher cancer risk [34]. In contrast, unsaturated fatty acids, which are typically present in vegetable oils, have antioxidant properties due to their bioactive compounds [34,35]. Hence, substituting saturated fatty acids with unsaturated ones contributes to the production of healthier foods.

As well as the total fat content, the control sample presented higher values for the crude fiber content and energy value. Adding guar gum and inulin to the emulsion produced a significant increase in the protein content compared to the other emulsions, although no statistically significant differences were observed when comparing the two oils.

Previous studies have reported that the choice of fat in sausage formulation significantly affects the protein content and that vegetable oils tend to generate higher levels of protein [36]. This phenomenon was, however, limited in our study, arguably due to the ingredients used in the emulsion. The different emulsion formulations showed different values for each of the parameters studied; for example, the combination of defatted chia flour and inulin presented the highest total carbohydrate and sugar content.

### 3.4. Fatty Acid Profile, Vitamin E and TBA Content

Table 7 shows the results obtained for the fatty acid profiles of the different reformulations of cured chorizo evaluated. The cured chorizo prepared with emulsified seed oils showed lower total fat content, with an increase in the polyunsaturated fatty acid content also being observed.

The control sample presented higher content of saturated (myristic, palmitic and stearic) and monounsaturated fatty acids (oleic acid). Replacing pork backfat with the vegetable oil emulsions resulted in increased content of polyunsaturated fatty acids, such as linoleic acid (Omega 6) and linolenic acid (Omega 3). The addition of chia oil also resulted in a higher concentration of linoleic acid, while adding pumpkin oil resulted in a higher concentration of linolenic acid. These results are consistent with previous findings for chia oil [37] and pumpkin oil [38].

Previous studies have also reported that the fatty acid composition is closely related to the ingredients used in product formulations [5]. In the case of our control sample, there was a predominance of oleic, stearic, palmitic and myristic fatty acids, which coincides with the research by [39]. 

We also studied the lipid oxidation activity (TBA) and vitamin E content in the cured chorizo (Table 8). Determining these is important to understand more about the product’s qualities and the correlations between them.

The TBA index, which is associated with a product’s lipid oxidation, revealed that the emulsions with chia oil were more prone to lipid oxidation than those produced with pumpkin oil. The deterioration of the sensory and nutritional quality of foods, regardless of their origin (animal or vegetable), is closely related to lipid oxidation, which is affected by both the internal and external conditions [40,41]. Low vitamin E content has also been found to favor oxidation, the effect of which varies according to the product composition. In the new reformulations, the lipid oxidation was found to decrease as the vitamin E content increased. The latter is more abundant in pumpkin oil, thus resulting in one of the emulsions with the lowest incidence of oxidation.

Vitamin E is known for its antioxidant capacity and is used in animal-based foods to improve its oxidation state and thus the quality of the meat [42]. In addition to enhancing the quality of foods, vitamin E also intervenes in protein–membrane interactions, signal transduction and gene expression [43] and DNA repair [44] and facilitates anti-inflammatory [45]. However, animals do not synthesize vitamin E and so must obtain it from their diets [46] underlining the importance of food that is rich in this compound.

### 3.5. Sensory Evaluation

**Figure 2:**
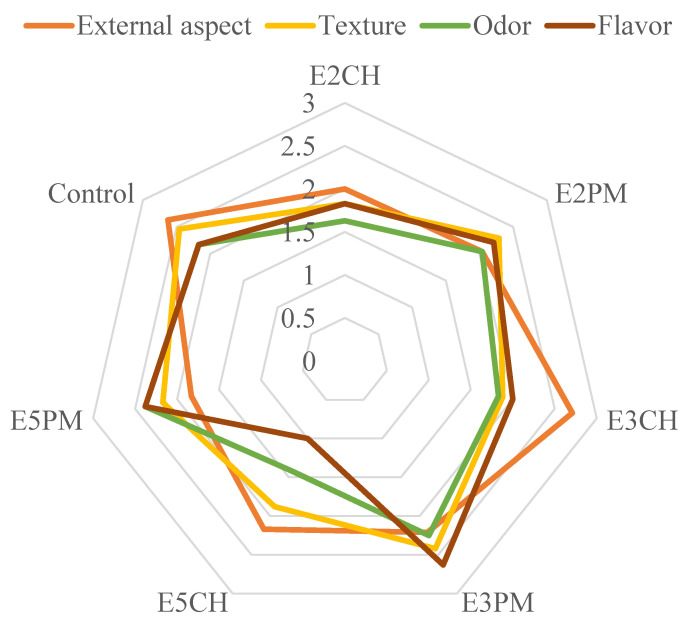
Results obtained for the sensory analysis (external appearance, texture, smell and taste) of the different cured chorizo reformulations evaluated. E2CH: chia oil emulsion with guar gum and inulin; E2PM: pumpkin oil emulsion with guar gum and inulin; E3CH: chia oil emulsion with defatted chia flour and inulin; E3PM: pumpkin oil emulsion with defatted chia flour and inulin; E5CH: chia oil emulsion with HPMC and xanthan gum; E5PM: pumpkin oil emulsion with HPMC and xanthan gum.

Figure 2 shows the results of the sensory analysis of the different formulations of cured chorizo evaluated. Significant differences were found according to the texturizer used in reformulating the product. The replacement of animal fat with the vegetable oil emulsions led to variations in the different parameters evaluated, confirming that fat influences the color, texture and flavor of meat products, and eliminating it can affect its sensory perceptions [5]. 

As regards the external aspect, the emulsion based on defatted chia flour, inulin and chia oil showed the highest preference values, surpassing even the control sample, which was the second most highly rated. Meanwhile, the formulation composed of an emulsion based on HPMC and pumpkin oil scored worst in terms of this sensory attribute.

The emulsions containing pumpkin oil were not well rated in terms of external appearance but were well evaluated, however, with respect to the other sensory attributes (texture, smell and flavor), presenting good values, with variations depending on the emulsion. The pumpkin oil emulsion with defatted chia flour and inulin obtained the highest values for flavor and texture, even surpassing the control sample. Additionally, the formulation containing pumpkin oil emulsion based on HPMC and xanthan gum obtained the best results for smell.

The addition of vegetable oils to meat products can improve their sensory evaluation [23,47], although high concentrations can compromise this improvement [48]. In our work, we found that this variation was also associated with the origin of the oil used, as different types of oil show different behaviors.

The worst ratings were observed in emulsions based on chia oil, which appeared to be due to its high content of linolenic acid (Omega 3), providing a characteristic fishy smell, particularly when subjected to high temperatures. In contrast, pumpkin oil, with a higher concentration of linoleic acid (Omega 6), presented higher ratings in the sensory analysis. Therefore, our research corroborates previous studies reporting that the adding oil in the production of meat products can influence their sensory evaluation, depending on the source of the oil used [49,50]. 

## 4. Conclusions

The use of vegetable oils in the form of emulsions is a viable alternative for pork backfat replacement in dry cured products. This replacement could be an important strategy to improve the nutritional and lipid characteristics of chorizo, while maintaining all of the sensory parameters above 0, which would ensure the positive consumer acceptance of this new product.

The main problem found in the reformulation is that the introduction of oil emulsions affects the physical parameters. More precisely, it affects the color, in terms of decreasing the luminosity and the red–green and yellow–blue tones in comparison with the control sample. For texture, the use of emulsions alters all texture parameters, especially the cohesiveness, which could cause the disintegration of the product.

The control sample shows higher content of saturated fatty acids (myristic, palmitic and stearic acids) and monounsaturated fatty acids (oleic acid). Replacing pork fat with textured vegetable oils results in an increase in the content of polyunsaturated fatty acids, such as linoleic acid (Omega 6) and linolenic acid (Omega 3). It can also be observed that the addition of chia oil results in a higher concentration of linoleic acid, while the addition of pumpkin oil results in a higher concentration of linolenic acid.

The TBA index, which is associated with the lipid oxidation of products, reveals that texturizations with chia oil have a greater propensity for lipid oxidation than those produced with pumpkin oil. It has also been observed that low vitamin E content favors oxidation, the effect of which varies depending on the composition of the product. In the new reformulations, a decrease in lipid oxidation is observed as the vitamin E content increases, which is more abundant in pumpkin oil, resulting, therefore, in one of the texturizations with the lowest incidence of oxidation.

The use of pumpkin oil and its high ratings position it as an alternative for the conversion of agri-food waste into a functional product and it could be used to reformulate countless products.

## Figures and Tables

**Table 1:** Ingredients used for the development of the different emulsions.

Sample	Oil (g)	Water (g)	Emulsifier
E1	100	100	6 g of beewax and 0.6 g of xanthan gum
E2	114	52.5	2.5 g guar gum and 5.25 g of inulin
E3	100	60	10 g of deffated chia flour and 30 g of inulin
E4	95	95	1.6 g of guar gum, 8 g maltodextrin and 1 g of xanthan gum
E5	120	78.6	2 g of hydroxypropyl methylcellulose (HPMC) and 1.2 g of xanthan gum

**Table 2:** Ingredients used for the development of the different emulsions.

Sample	Type of Oil	Emulsifier
E2CH	Chia	Guar gum + inulin
E2PM	Pumpkin	Guar gum + inulin
E3CH	Chia	Deffated chia flour + inulin
E3PM	Pumpkin	Deffated chia flour + inulin
E5CH	Chia	HPMC + xanthan gum
E5PM	Pumpkin	HPMC + xanthan gum

**Table 3:** Results obtained for the texture of the proposed oil emulsions.

Sample	Firmness (N)	Consistency	Cohesiveness (N)	Viscosity Index
E1CH	3.85 ^b^ ± 0.4	118.16 ^c^ ± 3.9	−4.94 ^b^ ± 0.2	−13.05 ^b^ ± 1.1
E1PM	8.44 ^b^ ± 0.5	107.6 ^cd^ ± 4.1	−10.62 ^c^ ± 0.4	−11.88 ^b^ ± 0.8
E2CH	6.46 ^b^ ± 0.3	209.7 ^b^ ± 5.5	−8.26 ^c^ ± 0.4	−21.47 ^b^ ± 1.2
E2PM	7.61 ^b^ ± 0.7	175.7 ^bc^ ± 6.7	−9.00 ^c^ ± 0.3	−18.04 ^b^ ± 1.3
E3CH	10.28 ^b^ ± 0.5	377.26 ^a^ ± 9.3	−17.2 ^c^ ± 0.2	−50.24 ^c^ ± 2.1
E3PM	11.34 ^b^ ± 0.7	344.75 ^a^ ± 2.5	−17.35 ^c^ ± 1.4	−49.26 ^c^ ± 2.7
E4CH	1.67 ^b^ ± 0.6	52.01 ^d^ ± 3.2	−1.89 ^b^ ± 1.3	−5.24 ^b^ ± 0.3
E4PM	1.79 ^b^ ± 0.4	65.15 ^d^ ± 3.7	−2.81 ^b^ ± 0.4	−8.02 ^b^ ± 0.6
E5CH	618 ^a^ ± 2.3	108 ^cd^ ± 3.9	2.05 ^a^ ± 0.2	50.73 ^a^ ± 4.1
E5PM	619 ^a^ ± 3.5	139 ^c^ ± 4.1	1.32 ^a^ ± 0.3	47.36 ^a^ ± 3.5
Control	613 ^a^ ± 4.1	267 ^ab^ ± 8.3	5.95 ^a^ ± 0.6	52.63 ^a^ ± 2.9

E1CH: chia oil emulsion with beeswax and xanthan gum; E1PM: pumpkin oil emulsion with beeswax and xanthan gum; E2CH: chia oil emulsion with guar gum and inulin; E2PM: pumpkin oil emulsion with guar gum and inulin; E3CH: chia oil emulsion with defatted chia flour and inulin; E3PM: pumpkin oil emulsion with defatted chia flour and inulin; E4CH: chia oil emulsion with guar gum with maltodextrin and xanthan gum; E4PM: pumpkin oil emulsion with guar gum with maltodextrin and xanthan gum; E5CH: chia oil emulsion with HPMC and xanthan gum; E5PM: pumpkin oil emulsion with HPMC and xanthan gum. Control: pork backfat. Different letters in the same column represent significant differences (*p* < 0.05) between samples.

**Table 4:** Results obtained for the color parameters of the different cured chorizo reformulations evaluated.

Sample	L*	a*	b*	C*
E2CH	32.89 ^b^ ± 2.03	18.31 ^b^ ± 2.37	19.42 ^b^ ± 2.85	26.72 ^b^ ± 3.55
E2PM	35.33 ^b^ ± 1.84	19.44 ^b^ ± 1.71	21.04 ^b^ ± 1.95	28.64 ^b^ ± 3.01
E3CH	33.52 ^b^ ± 2.35	16.59 ^c^ ± 1.63	19.28 ^b^ ± 2.87	25.43 ^b^ ± 2.44
E3PM	33.79 ^b^ ± 1.69	16.82 ^c^ ± 1.45	20.08 ^b^ ± 2.88	26.23 ^b^ ± 3.00
E5CH	33.74 ^b^ ± 1.68	12.37 ^d^ ± 3.40	12.81 ^c^ ± 1.93	17.96 ^c^ ± 3.04
E5PM	34.31 ^b^ ± 2.39	14.79 ^d^ ± 2.51	14.31 ^c^ ± 2.48	20.64 ^c^ ± 3.12
Control	43.87 ^a^ ± 1.78	23.52 ^a^ ± 1.77	29.10 ^a^ ± 3.09	37.44 ^a^ ± 3.19

E2CH: chia oil emulsion with guar gum and inulin; E2PM: pumpkin oil emulsion with guar gum and inulin; E3CH: chia oil emulsion with defatted chia flour and inulin; E3PM: pumpkin oil emulsion with defatted chia flour and inulin; E5CH: chia oil emulsion with HPMC and xanthan gum; E5PM: pumpkin oil emulsion with HPMC and xanthan gum. Different letters in the same column represent significant differences (*p* < 0.05) between samples.

**Table 5:** Results obtained for the texture parameters of the different cured chorizo reformulations evaluated.

Sample	Hardness (N)	Cohesiveness	Elasticity (mm)	Masticability (N·mm)
E2CH	278.79 ^b^ ± 21.45	0.46 ^ab^ ± 0.03	130.83 ^b^ ± 14.93	83.17 ^ab^ ± 9.98
E2PM	248.96 ^b^ ± 11.38	0.41 ^b^ ± 0.04	123.17 ^b^ ± 12.27	71.37 ^b^ ± 7.32
E3CH	359.39 ^a^ ± 11.09	0.48 ^a^ ± 0.03	172.89 ^a^ ± 15.86	95.38 ^a^ ± 13.43
E3PM	341.93 ^a^ ± 24.48	0.44 ^b^ ± 0.04	160.87 ^a^ ± 22.48	92.00 ^a^ ± 15.74
E5CH	206.06 ^c^ ± 25.16	0.44 ^b^ ± 0.02	92.23 ^c^ ± 14.69	44.59 ^c^ ± 8.48
E5PM	188.78 ^c^ ± 11.33	0.41 ^b^ ± 0.02	86.7 ^c^ ± 7.95	32.31 ^c^ ± 6.15
Control	207.48 ^c^ ± 11.3	0.51 ^a^ ± 0.02	107.4 ^c^ ± 7.72	65.73 ^b^ ± 20.47

E2CH: chia oil emulsion with guar gum and inulin; E2PM: pumpkin oil emulsion with guar gum and inulin; E3CH: chia oil emulsion with defatted chia flour and inulin; E3PM: pumpkin oil emulsion with defatted chia flour and inulin; E5CH: chia oil emulsion with HPMC and xanthan gum; E5PM: pumpkin oil emulsion with HPMC and xanthan gum. Different letters in the same column represent significant differences (*p* < 0.05) between samples.

**Table 6:** Results obtained for the nutritional analysis of the different cured chorizo reformulations evaluated.

Sample	E2CH	E2PM	E3CH	E3PM	E5CH	E5PM	Control
Moisture (%)	23.5 ^a^ ± 2.2	24.0 ^a^ ± 1.8	20.1 ^b^ ± 1.5	20.2 ^b^ ± 2.9	21.8 ^ab^ ± 2.6	22.5 ^ab^ ± 2.3	22.4 ^ab^ ± 2.0
Protein (g)	32.1 ^a^ ± 2.8	31.3 ^a^ ± 2.1	25.2 ^b^ ± 2.3	27.0 ^ab^ ± 1.8	28.2 ^ab^ ± 2.0	29.0 ^ab^ ± 1.7	26.2 ^b^ ± 2.9
Total fats (g)	31.6 ^c^ ± 3.1	30.8 ^c^ ± 1.7	33.6 ^c^ ± 2.0	30.3 ^c^ ± 2.3	39.1 ^b^ ± 2.1	37.0 ^b^ ± 1.8	49.3 ^a^ ± 2.8
Saturated fat (g)	7.1 ^b^ ± 0.9	9.1 ^b^ ± 0.7	8.0 ^b^ ± 1.0	8.9 ^b^ ± 0.9	8.1 ^b^ ± 0.5	9.1 ^b^ ± 1.1	18.6 ^a^ ± 1.2
Carbohydrates (g)	8.1 ^b^ ± 0.7	7.3 ^b^ ± 0.6	15.8 ^a^ ± 1.1	12.9 ^ab^ ± 1.1	3.7 ^c^ ± 0.5	2.9 ^c^ ± 0.6	2.7 ^c^ ± 0.2
Sugars (g)	4.1 ^b^ ± 0.1	3.3 ^b^ ± 0.2	8.9 ^a^ ± 0.1	5.8 ^a^ ± 0.4	1.6 ^c^ ± 0.2	0.6 ^c^ ± 0.1	0.5 ^c^ ± 0.0
Crude fiber (g)	1.9 ^b^ ± 0.2	2.1 ^b^ ± 0.1	2.5 ^b^ ± 0.3	2.6 ^b^ ± 0.4	2.8 ^b^ ± 0.0	1.8 ^b^ ± 0.3	5.8 ^a^ ± 0.1
Energy value (Kcal)	445 ^b^ ± 24	432 ^b^ ± 22	466 ^b^ ± 31	432 ^b^ ± 17	480 ^b^ ± 18	464 ^b^ ± 20	558 ^a^ ± 21

E2CH: chia oil emulsion with guar gum and inulin; E2PM: pumpkin oil emulsion with guar gum and inulin; E3CH: chia oil emulsion with defatted chia flour and inulin; E3PM: pumpkin oil emulsion with defatted chia flour and inulin; E5CH: chia oil emulsion with HPMC and xanthan gum; E5PM: pumpkin oil emulsion with HPMC and xanthan gum. Different letters in the same row represent significant differences (*p* < 0.05) between samples.

**Table 7:** Results obtained for the fatty acid profile of the different cured chorizo reformulations evaluated.

	E2CH	E2PM	E3CH	E3PM	E5CH	E5PM	Control
Myristic acid	0.51 ^c^ ± 0.04	0.53 ^c^ ± 0.04	0.66 ^b^ ± 0.03	0.62 ^b^ ± 0.02	0.51 ^c^ ± 0.03	0.53 ^c^ ± 0.04	1.45 ^a^ ± 0.05
Palmitic acid	13.70 ^c^ ± 1.2	18.60 ^b^ ± 0.9	14.80 ^c^ ± 1.5	18.7 ^b^ ± 1.1	12.5 ^c^ ± 0.9	18.8 ^b^ ± 1.4	24.1 ^a^ ± 2.1
Stearic acid	7.39 ^c^ ± 0.9	9.68 ^b^ ± 1.1	7.71 ^c^ ± 0.8	9.39 ^b^ ± 1.2	6.76 ^c^ ± 1.3	9.9 ^b^ ± 1.2	11.7 ^a^ ± 1.4
Oleic acid	20.80 ^c^ ± 2.4	33.80 ^b^ ± 2.8	24.50 ^c^ ± 3.1	36.2 ^b^ ± 4.1	19.3 ^c^ ± 2.9	35.5 ^b^ ± 4.3	50.1 ^a^ ± 5.2
Linoleic acid	20.20 ^b^ ± 1.7	34.70 ^a^ ± 2.9	17.20 ^b^ ± 3.1	30.8 ^a^ ± 2.7	18.4 ^b^ ± 2.0	32.2 ^a^ ± 3.1	7.97 ^c^ ± 3.2
Linolenic acid	35.10 ^a^ ± 3.2	0.55 ^b^ ± 0.15	32.50 ^a^ ± 4.1	1.92 ^b^ ± 0.09	40.5 ^a^ ± 2.1	0.76 ^b^ ±0.04	0.52 ^b^ ± 0.06

E2CH: chia oil emulsion with guar gum and inulin; E2PM: pumpkin oil emulsion with guar gum and inulin; E3CH: chia oil emulsion with defatted chia flour and inulin; E3PM: pumpkin oil emulsion with defatted chia flour and inulin; E5CH: chia oil emulsion with HPMC and xanthan gum; E5PM: pumpkin oil emulsion with HPMC and xanthan gum. Different letters in the same row represent significant differences (*p* < 0.05) between samples.

**Table 8:** Results obtained for the lipid oxidation activity and vitamin E content of the different cured chorizo reformulations evaluated.

Sample	TBA (mg MDA/kg)	Vitamin E (mg/kg)
E2CH	2.97 ^a^ ± 0.2	3.33 ^c^ ± 0.2
E2PM	0.70 ^b^ ± 0.1	5.75 ^b^ ± 0.2
E3CH	2.03 ^a^ ± 0.0	3.64 ^c^ ± 0.4
E3PM	0.49 ^b^ ± 0.0	7.39 ^a^ ± 0.3
E5CH	3.04 ^a^ ± 0.3	2.05 ^c^ ± 0.1
E5PM	0.66 ^b^ ± 0.1	10.6 ^a^ ± 0.4
Control	0.42 ^b^ ± 0.2	3.81 ^c^ ± 0.2

E2CH: chia oil emulsion with guar gum and inulin; E2PM: pumpkin oil emulsion with guar gum and inulin; E3CH: chia oil emulsion with defatted chia flour and inulin; E3PM: pumpkin oil emulsion with defatted chia flour and inulin; E5CH: chia oil emulsion with HPMC and xanthan gum; E5PM: pumpkin oil emulsion with HPMC and xanthan gum. Different letters in the same column represent significant differences (*p* < 0.05) between samples.

## Data Availability

The original contributions presented in the study are included in the article, further inquiries can be directed to the corresponding author.
